# Diagnostic challenges and treatment options in patients with solitary fibrous tumor: A single-center observational study

**DOI:** 10.3389/fsurg.2022.952463

**Published:** 2022-08-31

**Authors:** Andrej Ozaniak, Pavel Hladik, Robert Lischke, Zuzana Strizova

**Affiliations:** ^1^ Third Department of Surgery, First Faculty of Medicine, Charles University and University Hospital Motol, Prague, Czech Republic; ^2^ Department of Immunology, Second Faculty of Medicine, Charles University and University Hospital Motol, Prague, Czech Republic

**Keywords:** SFT treatment, SFT metastasis, SFT surgery, SFT surgical complications, soft tissue sarcoma, pseudosarcomatous lesions, sarcoma misdiagnosis, solitary fibrous tumor recurrence

## Abstract

**Introduction:**

Solitary fibrous tumor (SFT) is an extremely rare disease with a high misdiagnosis rate and a potentially malignant biologic nature. We have collected and analyzed data from 18 SFT patients to provide a deeper insight into this uncommon disease entity.

**Methods:**

In our study, 18 patients who had undergone surgery between April 2014 and December 2021 for the diagnosis of SFT were evaluated. The collected data for each patient included the location of the SFT, the preoperative diagnosis, the definitive histological diagnosis, the presence of postoperative complications, the time of recurrence, the time of systemic progression, the type of treatment, and the survival rate. The median follow-up was 36 months.

**Results:**

In three patients, the preoperative diagnosis did not correlate with the definitive histology of SFT. In patients with the limb location of SFT, no signs of recurrence nor distant metastases were seen within the study period. In total, 50% of the postsurgical complications were associated with the abdominal location of the SFT. In newly diagnosed SFT patients, two patients (20%) developed local recurrence, and the median time until recurrence was 22.5 months. Out of patients that were admitted and operated on for recurrent SFT, 67% relapsed, and the median time to relapse was 9.5 months. The systemic progression of the disease was observed in 33% of patients treated for recurrent SFT.

**Conclusion:**

In our study, the misdiagnosis rate was high and correlated with previously published studies. Postsurgical complications were associated with the extrathoracic location of SFT. The mainstay of SFT treatment remains radical surgery, although radiotherapy alone can significantly improve overall survival. Clinical trials are urgently needed to evaluate the potential effect of other treatment modalities, such as immunotherapy and targeted therapy, in SFT patients.

## Introduction

Solitary fibrous tumors (SFTs) are rare fibroblastic mesenchymal neoplasms that arise in various anatomic locations (1). Due to similarities to other soft tissue tumors, SFTs can often be difficult to diagnose and treat (2). The biological behavior of SFTs is uncertain; nevertheless, metastatic potential has already been observed (1). In previous studies, most SFTs were shown to be associated with an indolent clinical course but also displayed patterns of distant metastases in up to 40% of patients at 10 years of follow-up (3–5). Moreover, relapse-free survival in a follow-up period of approximately 20 years was reported to be less than 20% (5). Several risk classification models were created and eventually proven as accurate in predicting the risk of disease recurrence (6–8). The established risk factors mostly included disease-specific features, such as tumor size and location, mitotic count, and patient’s individual characteristics, such as age and sex (6–8).

In most cases, the size of SFTs ranges from 7 to 10 cm (1). Size and location are presumably by far the most important prognostic factors (4). In particular, size greater than 8 cm was associated with both local and distant recurrences. Depending on the size, the disease may exhibit nonspecific symptoms due to the compression of surrounding organs. However, most SFTs are painless and slow-growing (9). Extremely rare are paraneoplastic syndromes, such as Doege-Potter syndrome or Pierre-Marie-Bamberger syndrome (10). SFTs can be divided according to several criteria (1, 2). Conventional classification divides SFTs according to their location into intrathoracic (pleuropulmonary) SFTs, accounting for over 30% of the cases, intra-abdominal SFTs, SFTs of the head and neck (intracranial or extracranial) and SFTs of the soft tissues (1, 2). The importance of disease location in the patient’s prognosis was pronounced by multiple studies; however, the aggressive behavior of SFTs has been mostly associated with two SFTs locations, intrathoracic and retroperitoneal/intra-abdominal locations (4, 6, 11).

SFTs can also be alternatively classified according to their histological features (1, 2, 12). Interestingly, it has been shown that SFTs that lack malignant histological features in primary resection specimens may still acquire these features at the time of recurrence (13). The diagnosis of SFT is usually made upon a combination of imaging techniques, pretreatment biopsy, and histopathological evaluation (1). Here, we present our single-center experience in the treatment and management of this rare disease in 18 patients.

## Materials and methods

To analyze the clinicopathological features of SFTs, all patients who had undergone surgery between April 2014 and December 2021 for the diagnosis of SFT were evaluated. A total of 18 patients were enrolled in the study and provided written informed consent. The exclusion criteria for participation in the study were the presence of comorbid malignant conditions, unclear primary diagnosis, refusal to give informed consent, age <18, and pregnancy at the time of study initiation. However, none of the study participants met the exclusion criteria. Of the 18 study participants, 10 patients had newly diagnosed SFT without any previous treatment, 6 patients were admitted to our hospital for local recurrence of SFT, and 2 patients were admitted for a single metastasis of SFT. In recurrent/metastatic SFT, data regarding the previous SFT operation, including radicality of resection and perioperative/postoperative complications, were not evaluated. The patients’ clinicopathological data, including sex, age, preoperative and postoperative histology, and the provided therapy, were collected and analyzed. In patients who were surgically treated, the status of the resection margins was documented. The median age of our patients was 55 years, ranging from 33 to 80 years. The female:male ratio was 10:8. The collected data for each patient included the location of the SFT, preoperative diagnosis, definitive histological diagnosis, presence of postoperative complications, time of recurrence, time of systemic progression, systemic treatment given, and the survival rate. All patients’ data are summarized in Table 1. Statistical analysis was performed by GraphPad Prism 6 (GraphPad, La Jolla, CA) and Microsoft Excel (Microsoft for Windows, 2013). *P *< .05 was considered significant. For graphical presentation, Microsoft Excel and BioRender software were used.

**Table 1:** Data associated with the provided surgery and the outcome of the patients.

Clinical data of the patients	
Age, median (range)	55 (33–80)
Sex	
Female	10 (55.56%)
Male	8 (44.44%)
Primary site	
Intrathoracic	14 (77.78%)
Intra-abdominal	2 (11.11%)
SFT of soft tissues	2 (11.11%)
Operation	
Primary SFT	10 (55.56%)
Locally recurrent SFT	6 (33.33%)
Systemic SFT	2 (11.11%)
Radicality	
R0	17 (94.44%)
R1	1 (5.56%)
R2	0 (0%)
Complications	
Bleeding	3 (16.67)
Infection	1 (5.56%)
Without complications	14 (77.78%)
Local recurrence	
Primary SFT	2 (20%)
Recurrent SFT	4 (66.67%)
Systemic SFT	0 (0%)
Systemic progression	
Primary SFT	1 (10%)
Recurrent SFT	2 (33.33%)
Systemic SFT	0 (0%)
Follow-up, median (range)	36 (5–72)

SFT, solitary fibrous tumor.

## Results

### Preoperative misdiagnosis rate is high

**Figure 1:**
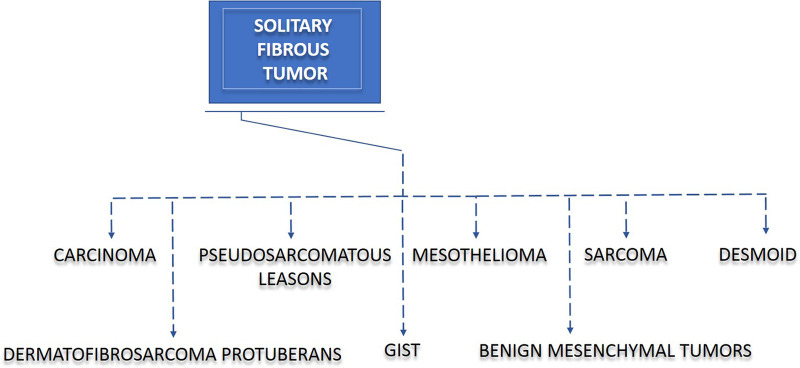
Solitary fibrous tumor (SFT) and disease overlap. SFT often causes difficulties in diagnosis and treatment. Due to a clinicopathological overlap, misinterpretation of other diseases may lead to incorrect diagnoses in patients with SFT. The diseases displaying the most similar patterns to SFT are sarcoma, gastrointestinal stromal tumor (GIST), mesothelioma, desmoid, benign mesenchymal tumor, dermatofibrosarcoma protuberans, carcinoma, and pseudosarcomatous lesions.

Owing to the complexity of histological features exhibited by SFTs, we aimed to evaluate the misdiagnosis rate in our study cohort. In three (16.67%) of our patients, the preoperative diagnosis did not correlate with the definitive histology of SFT. These three patients were primarily diagnosed with synovial sarcoma, pleural tumor, and peripheral nerve-sheath tumor (PNST). Thus, our data indicate that the preoperative diagnosis of SFT may cause difficulties, and therefore, the initial step in the differential diagnosis should contain the exclusion of other disease entities, such as sarcoma, gastrointestinal stromal tumor (GIST), and other diseases, as shown in Figure 1. Since biopsy highly contributes to the diagnostic process, in 12 (66.67%) of our SFT patients, a preoperative biopsy was performed. Six (33.33%) patients did not undergo preoperative biopsy, mainly due to the medical history of previous SFT at the same location.

### Intrathoracic SFTs are more frequent than extrathoracic SFTs

**Figure 2:**
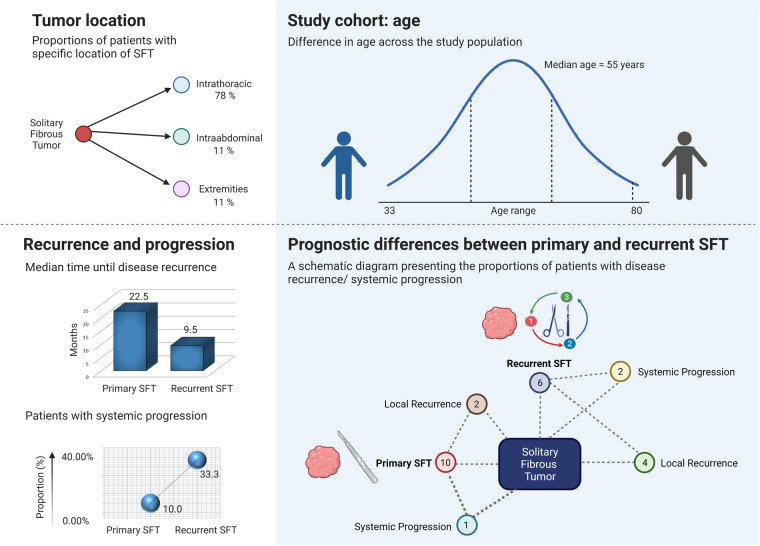
Prognostic variables and selected observations of the study cohort. The upper left section is a schematic diagram presenting the proportions of patients with intrathoracic (78%), intra-abdominal (11%), and limb location (11%) of solitary fibrous tumor (SFT). The upper right section presents the age range and age median of the study cohort. The lower left section shows that systemic progression of the disease was observed in 33.33% of patients who underwent surgery for recurrent SFT and in 10% of patients with newly diagnosed SFTs. Patients that were admitted and operated on for recurrent SFT tended to relapse earlier. The lower right section is a schematic diagram presenting the number of patients with disease recurrence/systemic progression. Out of 10 patients surgically treated for primary SFT, 2 (20%) patients developed local recurrence, and 1 (10%) patient presented with the systemic progression of the disease. Out of 6 patients who were admitted and operated on at our department for local recurrence of SFT, 4 (66.67%) patients presented with another episode of local SFT recurrence after being surgically treated, and 2 (33.33%) patients developed metastases.

The surgical management of SFT depends on the anatomic location of the tumor (12, 14, 15). Several locations of SFTs have been reported, including the salivary gland, larynx, orbits, liver, and pancreas (15). In our study of 18 SFT patients with a median age of 55 years, 14 (77.78%) patients had an intrathoracic location of the tumor, 2 patients (11.11%) had an abdominal location, and 2 (11.11%) patients had an SFT located in the lower extremities (Figure 2, upper left and upper right section, created with BioRender.com, No. JV244CL242). In patients with limb location of SFT, neither signs of recurrence nor distant metastases were observed within the study period. The same clinical course was observed in one of the patients with an abdominal location of SFT. The second patient with an abdominal location of SFT died of an unknown cause 5 months after the surgical treatment; however, there were neither signs of SFT recurrence nor systemic progression. In our study, the incidence of SFTs in the intrathoracic area was higher than that of those in other locations, which was in accordance with the study by Zhanlong et al. evaluating diverse SFT locations in 20 patients (16).

### Complete surgical excision leads to long-term survival

In SFT, surgery is the leading treatment option for localized disease (12, 15). The surgical management is similar to that of soft tissue sarcomas, and thus, obtaining negative resection margins is crucial (1, 12, 15).

In all 18 patients enrolled in our study, surgical resection was provided. Each surgery was performed by a specialist in oncologic surgery and assisted by a specialized thoracic/abdominal surgeon. Negative resection margins were achieved in 17 (94.44%) out of 18 patients. Although it has already been shown that a complete surgical excision leads to long-term survival, in one of our patients, the complete surgical resection was not accomplished (1, 12, 15). In this patient, the main tumor mass required a 10-cm resection of the sixth and seventh rib. However, with the further progress of the operation, multiple foci on the parietal, mediastinal, and diaphragmatic pleura were observed and led to a termination of the operation after a multidisciplinary assessment. In this patient, only the largest tumor mass, which was firmly fixed to the chest wall, was resected.

Even though local recurrence and seeding of the tumor on the peritoneal or pleural surface have been reported to have a significant association with the positive resection margins, in our study, the patient with positive resection margins did neither exhibit signs of recurrence nor distant metastases within the study period (26 months follow-up) (14). This was in contrast with the data of previously published studies (1, 12, 14).

### Surgical complications were mostly associated with the extrathoracic location of the SFT

Our previous observations prompted us further to investigate the surgical complications in our study cohort. A postsurgical complication occurred in 4 patients (22.22%) out of 18 study participants. Three complications were of hemorrhagic origin. One was an infectious complication. Two (50%) of the four documented complications were associated with the abdominal location of the SFT. The extrathoracic location of the SFT was shown by multivariate analyses to be an independent indicator of increased risk of disease recurrence (14). However, in our study, neither a tendency toward increased mortality nor toward disease recurrence was observed in abdominal/limb SFTs.

**Figure 3:**
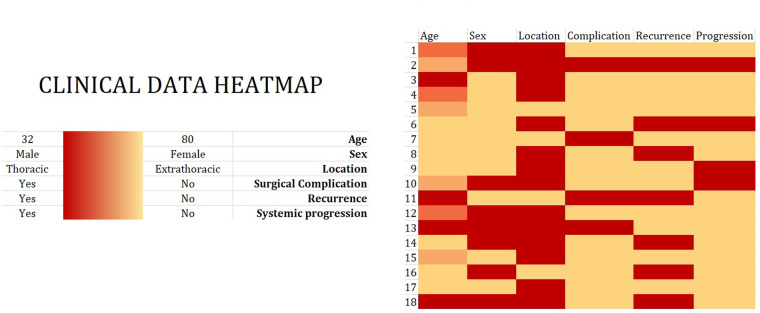
Clinical data heatmap. Eighteen patients underwent surgical treatment between April 2014 and December 2021 for the diagnosis of solitary fibrous tumor (SFT). Clinical data, including age, sex, tumor location, and disease recurrence/systemic progression, are visualized in a heatmap. The color intensity shows the magnitude of a selected phenomenon.

The incidence of surgical complications in each patient together with the clinicopathological data of the study participants is graphically presented as a heatmap (Figure 3).

### Patients treated for recurrent SFT tended to relapse more often and earlier than patients with newly diagnosed SFT

**Figure 4:**
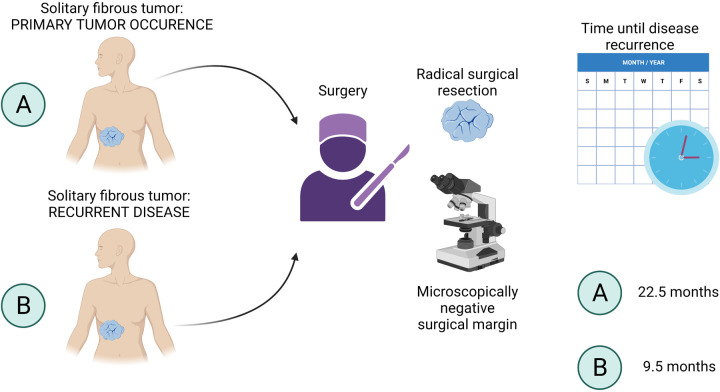
Time until disease recurrence. Patients with recurrent SFT (**B**) tended to relapse earlier (median 9.5 months, ranging from 5 to 19 months) than patients operated on for newly diagnosed SFT (**A**). The median time until disease recurrence in (**A**) patients was 22.5 months. SFT, solitary fibrous tumor.

In our cohort, we evaluated patients with local recurrence and patients with systemic progression to investigate the risk factors associated with these findings. Overall, the study participants with primary SFTs that were treated at our department had a low rate of systemic progression after surgical treatment. Out of 10 patients who underwent surgery for primary SFT, 2 (20%) patients developed a local recurrence and 1 (10%) patient presented with a systemic progression of the disease, as shown in Figure 2, lower right section. The systemic progression occurred 13 months after the surgery. Out of 6 patients who were admitted and operated on at our department for local recurrence of SFT, 4 (66.67%) patients presented with another episode of local SFT recurrence after being surgically treated, and 2 (33.33%) patients developed distant metastases (Figure 2, lower right section). The presence of metastases was associated with previous local recurrence. Two out of 18 patients who were admitted and operated on for metastatic disease did neither develop local recurrence nor distant metastases and remained disease-free in our study at 12 and 22 months. In patients who developed systemic progression within the study period, metastases of SFT were observed in the thorax (*n* = 2) or in both the thorax and abdomen (*n* = 1). The age of the patients did not correlate with the risk of local recurrence nor with the systemic progression. As shown in Figure 4 (created with BioRender.com, No. JW23XVB7UY), patients who were admitted and operated on for recurrent SFT (6 out of 18) tended to relapse more often (66.67%) and earlier (median 9.5 months, ranging from 5 to 19 months) than patients operated on for newly diagnosed SFT, where only 20% of the patients had local recurrence and the median time until disease recurrence was 22.5 months. Systemic progression of the disease was observed in 33.33% of patients who underwent surgery for recurrent SFT and in 10% of patients with newly diagnosed SFTs, as shown in Figure 2, lower left section. However, we admit that our study cohort is rather small due to the rarity of the disease entity, and therefore, further investigation is needed.

### Radiotherapy, targeted therapy, and immunotherapy reveal a great potential in the treatment of SFT

Chemotherapy can be given in both neoadjuvant and adjuvant settings; however, it has only limited efficacy in the treatment of SFT (1, 17). The superior efficacy of one approach over another (neoadjuvant/adjuvant) has not been reported. None of our patients received neoadjuvant chemotherapy. One of our patients received chemotherapy in an adjuvant setting after the failure of other therapeutic modalities. This patient died one year after the initial diagnosis.

Unfavorable tumor behavior serves as an indication of radiotherapy administration. Two of our patients received adjuvant radiotherapy after a local recurrence of the disease. One of the patients is still disease-free at 26 months after the initial diagnosis. The second patient died 39 months after the initiation of radiotherapy. None of our patients received targeted therapy or immunotherapy.

## Discussion

SFTs are rare mesenchymal tumors with a risk of local recurrence and a metastatic potential (1, 2). Although SFTs have been reported at almost every anatomic site, the intrathoracic location is the most prevalent one (18). SFTs belong to slow-growing tumors but may eventually cause pressure on the adjacent tissues (19). In our study, we shared our experience with 18 SFT patients.

Our data indicated that the preoperative misdiagnosis rate was high and corroborated that of previously published studies by Chu et al. and Kim et al. (20, 21).

In 17% of our patients, the preoperative diagnosis did not match the postoperative histology. Thus, our findings were in accordance with a previously published study on high misdiagnosis rates in SFTs (20). A compelling point of discussion was raised in a study by Hohenforst-Schmidt et al., revealing the importance of appropriate radiological examination as a part of the complex differential diagnosis (22). This, however, still presents a great hurdle since the imaging findings of SFTs are similar to those of other blood-rich tumors. Hence, to date, the risk of SFT misdiagnosis remains significant (22–24).

Biopsy highly contributes to the diagnostic process (1, 25, 26). In our study, the biopsy was not performed in patients with a previous medical history of SFT. This study group was directly operated on and included only those with the intrathoracic location of SFT. Saynak et al. suggested that a video-assisted thoracoscopic (VATS) biopsy may be considered an optimal approach to obtain a precise preoperative diagnosis (24). However, VAST biopsy is mostly superficial and thus, may not be sufficient in the diagnosis of SFT (27). We believe that VATS biopsy is particularly beneficial for the validation of the possible resectability of the tumor.

For histological verification, the standard diagnostic approach should include multiple core needle biopsies, possibly by using ≥14–16 G needles. Biopsy in deep-sealed tumors should be CT navigated and in superficial tumors, performed by a Tru-Cut needle. For superficial tumors ≤3 cm, excisional biopsy is the most convenient option (26, 28, 29).

In our SFT patients, we attempted to provide a complete surgical resection where possible. Negative resection margins were achieved in 94.44% of the patients. The need for obtaining negative resection margins stems from the fact that SFTs have uncertain biological behavior and a high rate of local recurrences (1, 4, 10).

Nonetheless, the surgical treatment may be difficult due to the abundant blood supplies that are often seen in SFTs (30). Wang et al. reported a case of SFT with vessel abnormalities in the tumor tissue, such as arteriovenous short circuits, which contributed to portal vein disease (17). Moreover, these abnormalities were neither obvious in the blood examination, electrocardiogram examination, nor in the chest radiograph examination before the laparotomy (17).

We observed a higher risk of local recurrence in patients who underwent surgery at our department for recurrent SFT than in patients who underwent surgery for primary SFT. In our study, the postoperative complications were associated with the extrathoracic location of the tumor. These findings, however, were limited by the number of study participants and required verification by further research. In addition, previous studies have demonstrated a tendency toward increased mortality in abdominal SFTs as compared to the SFTs in the limbs (14). This has not been observed in our study.

The late presentation of abdominal SFTs was discussed as a factor contributing to a higher mortality (14). We, however, also comment on the fact that providing a wide surgical excision and obtaining negative surgical margins is far more challenging in the abdomen than in the limbs.

Radiotherapy can be given as both neoadjuvant and adjuvant treatment (1). However, both adjuvant radiotherapy and chemotherapy are not routinely required in SFTs. In our experience, the incorporation of radiotherapy in SFT treatment should be considered in each patient who has undergone surgical excision without achieving negative surgical margins. In our study, two patients were given adjuvant radiotherapy, with only one of these patients being alive at the study termination. We highly support the study by Haas et al. on the efficacy of radiotherapy in sarcoma patients and patients with sarcomatous lesions (31). Moreover, studies have reported a promising efficacy of systemic therapies, such as bevacizumab, a humanized recombinant antibody against vascular endothelial growth factor (VEGF), together with temozolomide, an alkylating chemotherapeutic in SFT patients (32). Both pazopanib, an anti-VEGF receptor agent, and sunitinib, a tyrosine kinase inhibitor, have also shown potential in SFT treatment (33, 34).

Immunotherapy has not been approved for SFT so far. However, a single case report of a patient treated with an anti-PD-1 checkpoint inhibitor has shown remarkable results (35). The efficacy of different immunotherapies, thus, remains to be clarified.

## Conclusion

SFTs are diagnostically challenging malignancies with a high rate of misdiagnoses. Establishing the correct diagnosis requires a complex integration of clinical and histopathological features of the tumor, together with ruling out more common disease entities. Only a wide differential diagnosis excluding other potentially malignant tumors, such as soft tissue sarcomas, carcinomas, and/or GISTs, leads to accurate treatment selection.

The mainstay of SFT treatment remains radical surgery, where obtaining negative resection margins is the most important factor preventing the disease recurrence.

While radiotherapy alone can significantly improve the overall survival of patients, we believe that more therapies, mainly targeted therapy and immunotherapy, should become a part of the sophisticated therapeutic scheme in SFT (14, 19). Currently, a global consensus on the treatment SFTs is lacking and randomized clinical trials need to be designed for these rare disease entities. A multidisciplinary team approach should prevent the false management of these tumors.

## Data Availability

The authors are happy to share the raw data supporting the conclusions of this article on request to the corresponding author.
